# Relapsed acute myeloid leukemia presenting as conjunctival myeloid sarcoma: a case report

**DOI:** 10.1186/s12886-022-02286-1

**Published:** 2022-02-10

**Authors:** Joong Hyun Park, Yengwoo Son, Joon Young Hyon, Ji Yun Lee, Hyun Sun Jeon

**Affiliations:** 1 Department of Ophthalmology, Seoul National University Bundang Hospital, Seongnam, Republic of Korea; 2 Department of Ophthalmology, Seoul National University College of Medicine, Seoul, South Korea; 3 Division of Hematology-Oncology, Department of Internal Medicine, Seoul National University Bundang Hospital, Seongnam, South Korea

**Keywords:** Acute myeloid leukemia, Conjunctival myeloid sarcoma, Radiotherapy, Relapse, Case report

## Abstract

**Background:**

Conjunctival myeloid sarcoma (MS) as an isolated presentation of acute myeloid leukemia (AML) relapse is rare. Here, we report a case of unilateral conjunctival MS revealed as a sign of AML relapse.

**Case presentation:**

A 50-year-old man with a history of AML in remission visited our clinic presenting with a left conjunctival injection persisting for 1 month. Diffuse subconjunctival thickening with conjunctival vascular engorgement was observed. Ultrasound biomicroscopy revealed a hyper-reflective, thickened conjunctiva in his left eye. During the incisional biopsy, the lesion was strongly attached to the underlying sclera; histopathologic examination revealed infiltration of leukemic blasts. The relapse of AML was confirmed by a successive bone marrow biopsy. The ocular lesion disappeared after allogeneic peripheral blood stem cell transplantation (PBSCT) and concomitant salvage radiotherapy on the left eye. The patient has remained in remission for 3 years after allogeneic PBSCT.

**Conclusions:**

Incidental conjunctival lesions can indicate AML relapse in patients treated earlier for AML. An ophthalmologist may have a role in the early detection of AML when a patient presents with an atypical conjunctival lesion.

## Background

Myeloid sarcoma (MS) is an extramedullary solid tumor composed of immature myeloblasts and other granulocytic precursor cells with or without maturation occurring at sites other than the bone marrow (BM) [1]. The Society of Hematopathology and European Association for Hematology categorized MS according to the presence and stage of underlying leukemia at the time of diagnosis of MS as follows: (1) isolated MS; (2) MS with concurrent acute myeloid leukemia (AML); (3) extramedullary relapse of AML including relapse in the post-transplant setting; and (4) blast phase/transformation of a myeloproliferative neoplasm or chronic myelomonocytic leukemia [2]. Ocular and adnexal MS manifests most commonly in orbit, followed by the lacrimal glands or extraocular muscle; however, the conjunctiva is a rare site of involvement [3]. In the literature, there is a lack of long-term follow-up of conjunctival MS with concurrent AML, and the longest follow-up was 18 months [4]. Herein, we describe a case of conjunctival MS that was found to be a relapse of AML without any other systemic signs, with the longest follow-up in the literature.

## Case presentation

**Fig. 1:**
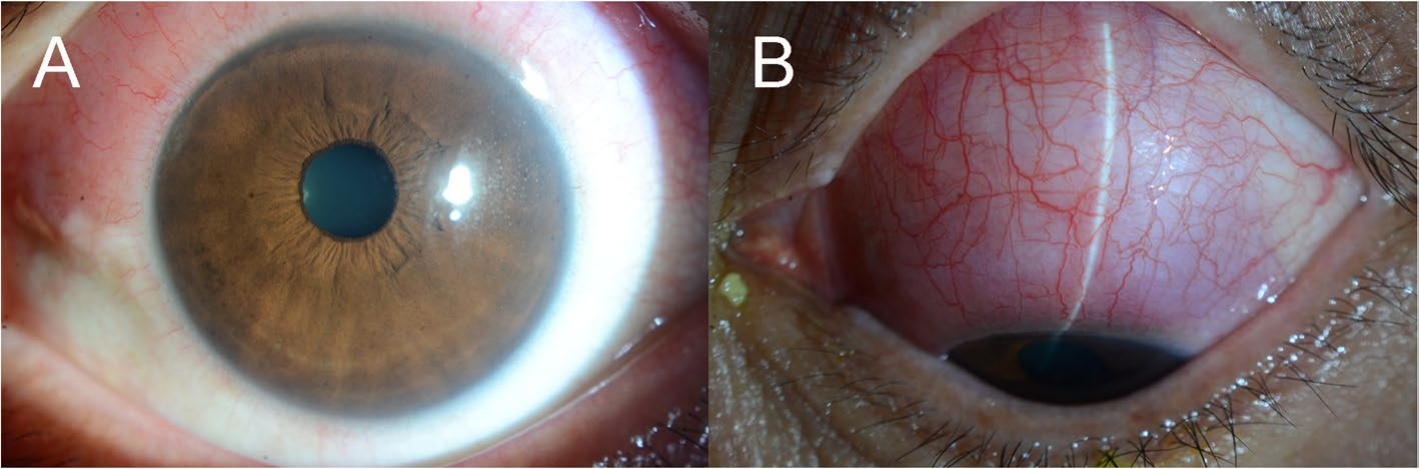
Anterior segment photography demonstrates diffuse injection and thickening of the conjunctiva (from 9 to 2 o’clock) in the left eye (**A**, **B**)

A 50-year-old man visited our clinic complaining of an redness with a foreign body sensation in his left eye that lasted for 1 month. He had a medical history of *NPM1*-mutated AML, and was in complete remission after receiving chemotherapy (CT) 2 years ago. Slit lamp examination revealed diffuse conjunctival thickening with injection in the left superior conjunctiva extending for approximately 5 clock hours (from 9 to 2 o’clock), with no abnormal findings in the cornea, anterior chamber, iris, and lens (Fig. 1). No remarkable lesions were observed on fundus examination. Topical 0.5% levofloxacin and 1% prednisolone acetate were administered empirically; orbit MRI and ultrasound biomicroscopy were planned. After 1 week, the conjunctival lesions did not show any improvement.

### Investigations

**Fig. 2:**
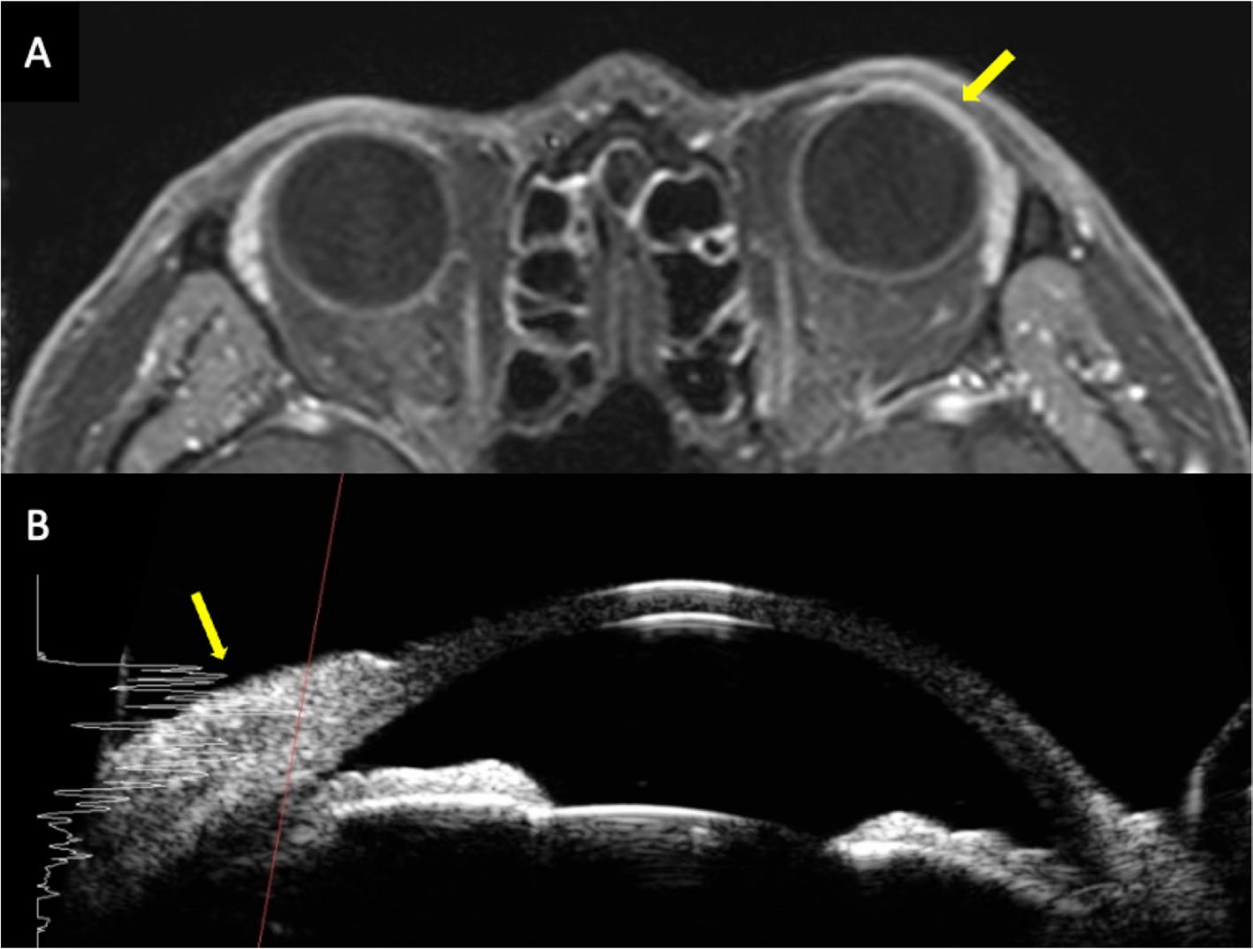
Orbit MRI (**A**) and ultrasound biomicroscopy (**B**) show linear thickening and hyperreflectivity (yellow arrows)

**Fig. 3:**
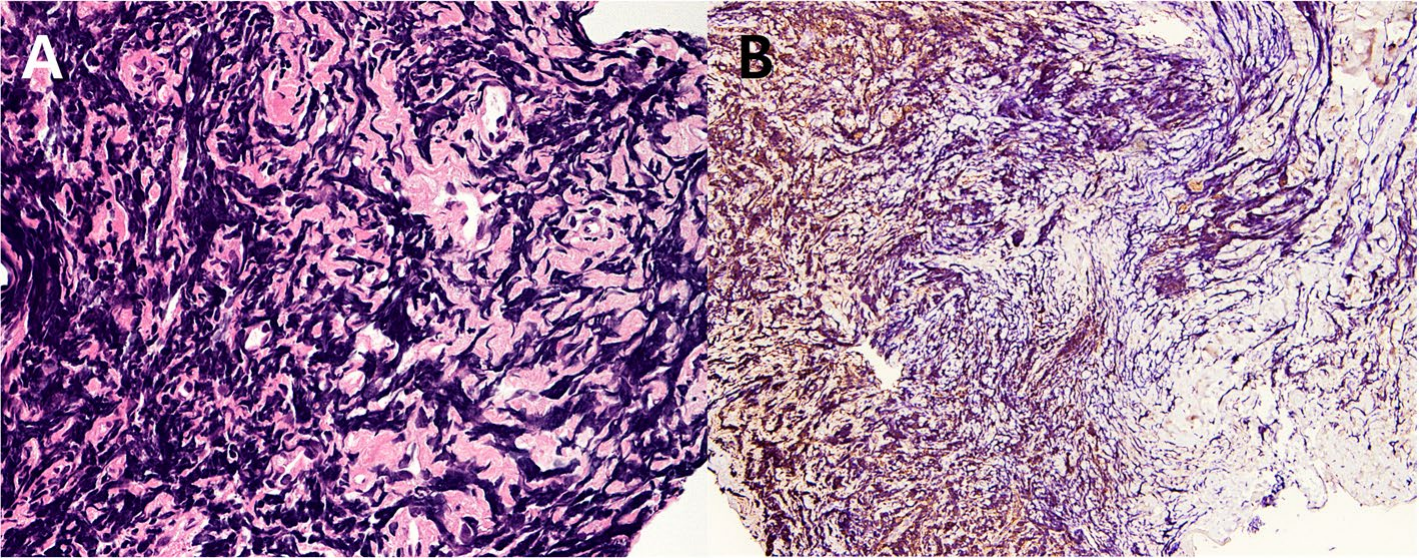
Histopathologic characteristics of the conjunctival myeloid sarcoma. **A** Hematoxylin and eosin (H&E) stained section revealed a population of pleomorphic cells, a few with angulated nuclei and scant cytoplasm (H&E, × 400); **B** Immunohistochemical staining of myeloperoxidase (MPO) shows focal positivity (brown), suggesting the presence of myeloblasts (MPO, × 100)

Orbital MRI demonstrated linear thickening and enhancement of the left conjunctival area, suggestive of conjunctival lymphoma or inflammation. Ultrasound biomicroscopy also revealed linear thickening of the conjunctiva along with firm adhesion (Fig. 2). Incisional biopsy of the conjunctival mass was performed considering the medical history and appearance of the lesion. During the incisional biopsy, the subconjunctival tissue was strongly attached to the sclera, and the medial rectus showed ill-defined margins. A cautious biopsy was performed to preserve the normal anatomical structure against insults caused by surgery. The immunohistochemical profile was positive for Ki-67, CD33, CD117, and myeloperoxidase, suggesting MS (Fig. 3).

The patient was referred to the hemato-oncology department for further systemic evaluation. BM biopsy was performed immediately; cellularity of 21–30%, hypocellular for age, and an increase in CD117 immature cells were observed. It also showed an increase in blasts (6.4% of total nucleated cells), and the relapse of AML with concurrent conjunctival MS was confirmed.

### Treatment

The patient was treated with anti-leukemic reinduction CT (mitoxantrone, etoposide, and cytarabine), concomitant allogeneic peripheral blood stem cell transplantation (PBSCT) for the systemic relapse of AML, and salvage radiotherapy (RT) for 3 weeks (24 Gy/12 cycles) for the conjunctival MS.

### Outcome and follow-up

**Fig. 4:**
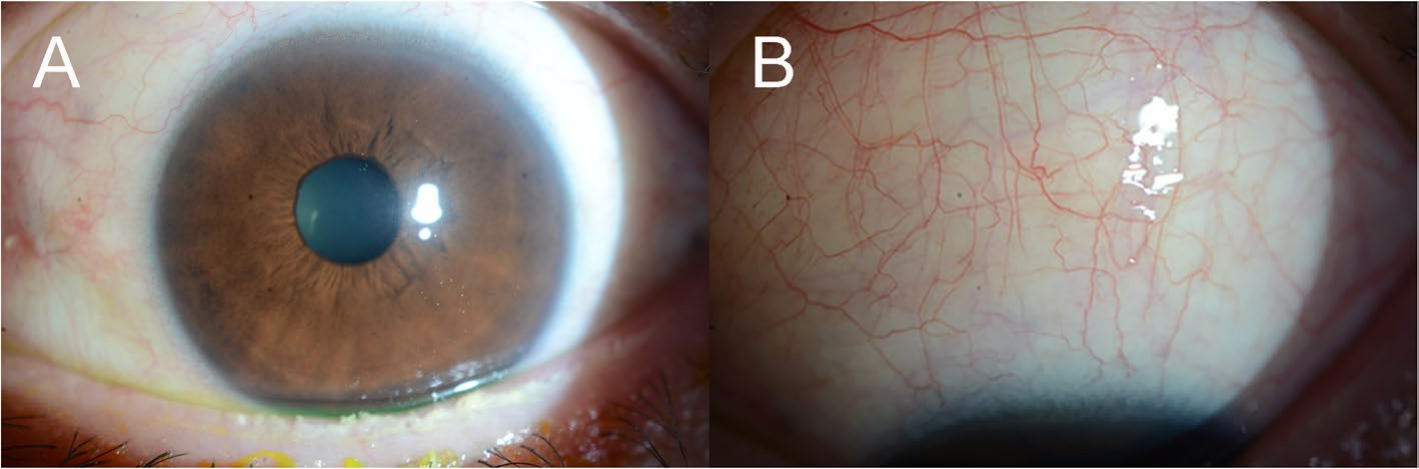
The previous conjunctival lesion in the left eye resolved 3 months after chemotherapy and salvage radiotherapy for 3 weeks (24Gy/12 cycles) (**A**, **B**)

**Fig. 5:**
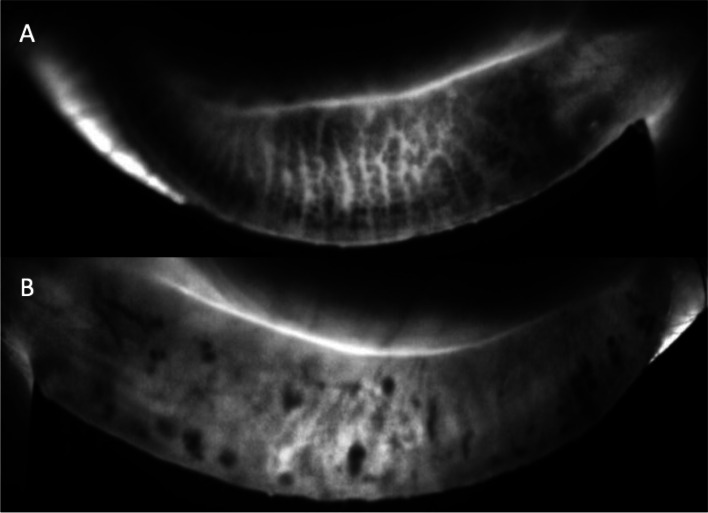
Compared to the meibomian glands of the contralateral right eye (**A**), the left eye shows diffuse severe dropouts of meibomian glands in the contact meibography 3 months after localized radiotherapy (**B**)

After 3 months, the previous conjunctival mass in the left eye had resolved (Fig. 4) and was maintained after 3 years. However, he complained of dry eye symptoms in his left eye. Slit lamp examination revealed obstruction of the meibomian gland orifices, and moderate punctate epithelial erosions were seen. The LipiView II interferometer (TearScience Inc., Morrisville, North Carolina, USA) has demonstrated severe meibomian gland dropout in the left eye compared to the right eye (Fig. 5). After 3 years of follow-up, the conjunctiva remained clear and did not show any recurrence, and the patient remained in a favorable general condition.

## Discussion and conclusions

MS is often accompanied by hematologic malignancies, including AML, myeloproliferative neoplasm, myelodysplastic syndrome, and the blast phase of chronic myeloid leukemia. AML is the most common concomitant systemic disease, and MS has been reported to occur in 3–9% of all AML cases [5]. In ophthalmic practice, the orbit is the most common site of ocular and adnexal MS, and recently Al Semari et al. reported two cases of orbital MS without evidence of AML and emphasized the prompt diagnosis of isolated, primary, or non-leukemic MS is crucial for effective clinical management [6]. MS occurring in the conjunctiva is rare, and only a few cases have been reported [7–13]. Among them, only three cases have been reported so far as unilateral conjunctival MS occurring as a sign of relapsed AML in adults [11–13]. In our case, the lesion appeared as a unilateral conjunctival mass extending for about 5 clock hours, which was clinically similar to that reported by Meel et al. [4]. It masqueraded as nodular scleritis or conjunctival lymphoma, which made making an an accurate diagnosis more difficult. Although the patient was in AML remission status, a conjunctival biopsy was performed considering the patient’s previous systemic medical history. The lesion was firmly attached to the sclera during the incisional biopsy, and a hard texture was observed. It was different from that of conjunctival lymphoma; therefore, we expected an unusual diagnosis. After revealing the conjunctival MS, the patient was referred to the hemato-oncology department. BM biopsy was performed, and relapse of AML with concurrent conjunctival MS was finally diagnosed.

The presentation timing of MS varies: 25% occurs as isolated MS without AML, 15–35% occur concurrently with the diagnosis of AML, and up to 50% occur after or at the time of AML relapse [14, 15]. Of the eight cases of AML reported having conjunctival MS, four cases presented with an initial manifestation of AML, and the remaining four cases manifested with relapse of AML (Table 1) [4, 7, 16–21]. In our case, conjunctival MS was the initial sign of AML relapse. We detected relapsed AML in the early period, which presented a 1-month history of conjunctival injection before the biopsy was performed, while the study by Meel et al. reported a 5-month history of a conjunctival mass [4]. The prognosis of MS is poor and varies according to several factors, including the type of hematologic disorder, age, and the location of involvement [15, 22]. The median survival period of isolated MS was reported to be approximately 36 months, and it becomes shorter by 6–14 months when it is accompanied by AML [23]. Although several factors may influence the prognosis, the favorable results of our patient are due to the early detection of AML relapse.

**Table 1:** Cases of conjunctival myeloid sarcoma included in the literature review until 2021

Author (Year)	Sex/Age	Laterality	Location	Clinical manifestation	Treatment	Outcome	Follow-up
Font et al. (1985) [16]	F/28	Bilateral	Perilimbal	Relapse	CT + RT	CR	Disease free at 7 months
Lee et al.(1985) [17]	M/57	Bilateral	Palpebral, diffuse (giant papillary reaction)	Initial manifestation	NA	NA	NA
Mansour et al. (1985) [18]	M/28	Bilateral	Caruncular and diffuse palpebral mass	Relapse	NA	NA	NA
Tsumura et al. (1991) [19]	F/29	Left	Focal bulbar	Relapse	CT	CR of conjunctival lesion	Died at 2 months
Douglas et al. (2002) [20]	F/42	Right	Focal limbal	Initial manifestation	CT	CR	Disease free at 1 month
Fleckenstein et al. (2003) [21]	F/73	Bilateral	Diffuse bulbar	Initial manifestation	CT + RT	CR	Died at 5 months
Hong et al. (2011) [7]	F/10	Right	Focal bulbar	Relapse	CT + STC	CR	Disease free at 16 months
Meel et al. (2019) [4]	F/28	Left	Perilimbal	Initial manifestation	CT	CR	Disease free at 18 months
Current case (2021)	M/50	Left	Focal limbal and bulbar	Relapse	CT + RT, PBSCT	CR	Disease free at 3 years

*CR* complete regression, *CT* Chemotherapy, *NA* Not available, *PBSCT* Peripheral blood stem cell transplantation, *RT* Radiotherapy, *STC* Stem cell transplantation

The consensus for MS treatment is not well-established owing to the lack of randomized control trials. Chemotherapy is currently the mainstay of treatment, including isolated MS; other treatment options are RT and surgery. He et al. found that neither RT nor surgery delayed the transformation of MS to AML without systemic CT [11]. Some studies have reported a higher AML progression rate with isolated MS in patients who received only localized therapy compared to those who received systemic CT [15, 24]. Several studies have reported beneficial outcomes of standard CT combined with localized RT [21, 25], and it could be specifically effective in reducing the size of the mass. However, Lan et al. reported that survival rates of MS patients were not different between the CT group and the combined RT and CT group [15]. Sharma et al. also suggested that leukemic infiltration of the conjunctiva shows good responses to CT, and additional RT is not required [26]. As tumor invasion of the medial rectus muscle was observed during the biopsy, we decided to perform RT in addition to CT. Three months after treatment, the conjunctival MS had resolved. However, the patient complained of discomfort in his left eye, and diffuse drop out of the meibomian glands was observed on meibography. As the morphology of the meibomian glands of the contralateral right eye was intact, we concluded that it was a complication of RT. The patient had symptoms and signs of dry eye disease with superficial punctate keratitis of the left eye and has been treated with topical eyedrops, including artificial tears, 0.05% cyclosporine, and warm compressions. As he also received allogeneic PBSCT, ocular graft-versus-host disease may be one of the causes of dry eye. However, considering that he had unilateral dry eye with diffuse meibomian gland dropout, RT could have affected the patient’s status; therefore, concomitant RT should be carefully considered as a treatment for ocular MS and management of meibomian gland dysfunction should also be performed after concomitant RT.

This is a rare case of conjunctival MS presenting as the first sign of AML relapse, and prompt diagnosis and treatment are necessary to confer a better prognosis for this life-threatening disease. Incidental conjunctival lesions should be considered a warning sign indicating ocular involvement of the systemic disease, especially in patients with a history of cancer.

## Data Availability

The datasets included in the current study are available upon reasonable request.

## Abbreviations

MS: Myeloid sarcoma
AML: Acute myeloid leukemia
PBSCT: Peripheral blood stem cell transplantation
BM: Bone marrow
CT: Chemotherapy
RT: Radiotherapy
